# Do Moral Judgments in Moral Dilemmas Make One More Inclined to Choose a Medical Degree?

**DOI:** 10.3390/bs13060474

**Published:** 2023-06-05

**Authors:** Elena Druică, Toni Gibea, Rodica Ianole-Călin, Emanuel Socaciu

**Affiliations:** 1 Department of Applied Economics and Quantitative Analysis, Faculty of Business and Administration, University of Bucharest, 030018 Bucharest, Romania; elena.druica@faa.unibuc.ro; 2 Department of Philosophy and Social Sciences, Faculty of Management, Bucharest University of Economic Studies, 010374 Bucharest, Romania; toni.gibea@man.ase.ro; 3 Faculty of Philosophy, University of Bucharest, 060024 Bucharest, Romania; emanuel.socaciu@filosofie.unibuc.ro

**Keywords:** moral judgments, medical education, sacrificial moral dilemmas, medical ethics, academic choice, empathy

## Abstract

The role of moral intuitions and moral judgments has become increasingly prominent in educational and academic choices. The present research aims to examine if the moral judgments elicited in sacrificial trolley dilemmas have a distinct pattern for the decisions made by junior medical students, in comparison to those of senior high school students. We work with this sample because it represents the population out of which medical students are recruited in the case of Bucharest, Romania. Our findings show that moral judgments are indeed a significant predictor for a respondent’s status as medical students. This result, albeit with limitations, bears multiple practical implications, from developing empirically informed medical ethics courses in medical schools to evidence-based policy designs which consider factors such as morality alongside financial outcomes and incentives.

## 1. Introduction

Many modern debates are built around questions looking at the optimal combination between developing moral judgment competencies and strengthening professional skills, in various contexts of human development, to increase individual and societal wellbeing [1]. There are significant differences between fields, with salient research focusing on business [2], public administration [3], medicine and law enforcement [4], and, more recently, artificial intelligence [5,6]. One area of particular interest is to understand the entering point into these careers, namely the determinants of academic choice. Among the most frequently considered factors, we find the expected economic returns of a future profession [7,8], personality traits or the quality of available information [9], the current labor market challenges [10,11], student loans bubble [12], increased competition between higher education institutions [13,14], academic reputation or educational marketing [15,16], or intrinsic motivations [17]. When applying this extensive list to careers highly dedicated to human service and in high demand (such as medical practice), and to the new generation’s beliefs, it becomes a stringent policy priority to correctly delimitate motivational influences [18,19]. For instance, a recent systematic review [20] has classified medical students’ motivations into three categories: (i) scientific factors (e.g., cognitive interest, research opportunities, the intellectual satisfaction of working in a cutting-edge field), (ii) societal factors (e.g., high status and income, job security, family encouragement and tradition), and (iii) humanitarian factors (e.g., desire to help others or to give back to the community). It is noteworthy to mention that while the scientific motivations are more prevalent in affluent countries and the societal motivations are more prevalent in developing countries, the humanitarian factors are present to similar degrees in both categories. This finding is compatible with the view that the medical profession is, to a large degree, a vocational one [21,22,23,24,25]. In this vein, being a doctor is often described in terms of authority, commitment, and duty [26], all of them indicative ingredients of an implicit moral dimension. Similarly, both a conscious and an unconscious sense of helping other people is recognized as a key driver of choosing a medical degree [27,28]. This acknowledgement of the unconscious factors extends the focus of this research field to new variables [29], such as non-cognitive abilities [30,31] and personality traits [32], next to the underexplored moral reasoning and moral orientation [33].

Targeting the morality dimension, we observe that, in general, there is consistent evidence that personal, economic, and social decisions are influenced by our moral judgments or beliefs: energy-saving behavior correlates with consumers’ environmental belief and attitude [34], green buying decisions correlate with ethical beliefs [35], the denial of the moral status of animals correlates with meat consumption [36], and implicit beliefs about moral character influence trust recovery [37].

Thus, based on this solid background, the current paper aims to investigate if moral judgements increase the likelihood of one choosing a medical career. To that purpose, we employed the powerful tool of philosophical ethical dilemmas, commonly used in practice as a reliable way of measuring how lay people make moral judgments [38,39,40,41,42,43], but also as a type of off-the-shelf solution for undecidable life problems. We designed an exploratory study testing whether first-year medical students are more prone to make the kind of moral judgments that would be derived from the core ethical commitments of medicine when are faced with traditional moral dilemmas, by comparison to ordinary high school students. Our methodological approach is innovative in terms of design but it also aligns with the extant literature connecting moral decision-making with various professional behaviors [44] and, implicitly, instilling relevant insights for educators (especially health and medical sciences) [45,46] and strategic policy makers.

In the next section, we present the dilemmas that we used to test the moral judgments of high school and medical students. Then, we outline the data, the method of analysis and the results, while in the final section we discuss the results, along with some limitations of the study and several practical implications.

## 2. Materials and Methods

### 2.1. Data

We collected data from two different groups: high school students and medical students in their first year of study. Data was gathered during the first three weeks of the academic year to minimize contagion effects from courses or seminars on medical ethics topics. The University of Bucharest does not require ethical committee approval for questionnaire-based research.

For the high school group, the surveys were distributed by several philosophy professors who did not discuss beforehand with their students about ethics or other moral topics. For the second group, junior students in medicine, the surveys were distributed before a class on cellular biology. Participation was voluntary and anonymous, and no monetary incentives were offered. We collected our data using a pen and paper questionnaire.

### 2.2. Measurement

As measurements, we departed from the more common psychometric approach [33] by employing a type of moral dilemmas, often labeled ‘sacrificial’, to examine the participants’ moral judgments: the trolley (TD) and the footbridge dilemma (FD), together with two additional modified versions (MTD and MFD). The trolley dilemma was devised by Philippa Foot [47] as a thought experiment meant to illustrate the Doctrine of Double Effect. We framed the dilemma as follows:

*“You are near a railway track when you suddenly see a runaway trolley. Further down its course five persons are tied up to the tracks. If you choose to do nothing, all five will be killed by the trolley. Luckily, there is a switch near you. If you activate the switch the trolley will be redirected to a secondary track. But you know that on that second track another person is tied up, who would be killed by the trolley if you activate the switch. You know that what happens next with all these people is the result of your decision.”*

Judith Thomson [48] proposed a different thought experiment to highlight the fact that intended sacrifice is not permissible. This thought experiment is usually referred to as the footbridge dilemma and we framed it in the following way:

*“You are near a fat man on a footbridge that crosses a rail. You suddenly see that a runaway trolley is heading towards your direction and threatens the life of 5 people who were tied up to the tracks. The only thing you can do, in order to save the lives of these 5 people, is to push the fat man over the footbridge, in front of the trolley, sacrificing his life but stopping the trolley.”*

Previous research [38] explored how uncontrolled emotional impact and spatial proximity, alongside others, impact upon moral judgments by controlling different aspects of the sacrificial dilemmas. In a similar fashion, we modified the above-mentioned two dilemmas. In the MTD scenario, we told the participants to imagine that the switch is placed near the person who is tied up to the railways, highlighting the fact that if they decide to sacrifice it, to save the other five persons, they will see in front of them how that one person is run over by the trolley. In the MFD scenario, we told the participants to imagine that they are in an office, miles away from the footbridge, and that while they are drinking their coffee, they see on the monitors that a trolley has become out of control and is heading towards five tied-up people. They were told that the only thing they could do to save the 5 people is to sacrifice the fat man by pushing a button which operates a trap under the footbridge (see Appendix B).

Each scenario was placed on a different page, with a graphic representation of the dilemma. For each scenario, the participants had to answer four different questions: (i) factual question: ‘Would you activate the switch/push the fat man/push the button and kill 1 person in order to save 5’; (ii) acceptable: ‘Is it ok to activate the switch/push the fat man/push the button and kill 1 person in order to save 5?’; (iii) morally permissible: ‘Is it morally permissible to activate the switch/push the fat man/push the button and kill 1 person in order to save 5?’; (iv) moral duty: ‘Is it a moral duty to activate the switch/push the fat man/push the button and kill 1 person in order to save 5?’. For every question, the respondents had to choose between a yes and no answer.

### 2.3. Methods

Given our intention to classify our respondents according to their responses to the moral dilemmas, we used logistic regression to check to what extent the choice made by the respondents in each of the four scenarios predicts whether the respondent is a high school student or a medical student. Our model provides an estimation of the log of the odds of being a medical student compared to a high school student, according to Equation (1).

Log(odds) = ß_0_ + ß_1_ * TD + ß_2_ * MTD + ß_3_ * FD + ß_4_ * MFD + ß_5_ * Controls + e (1)

To avoid overfitting, we conducted cross-validation using 70% of the original data as the train set, and the remaining 30% as the test set. In addition, we conducted a sensitivity analysis, aiming to explore the adjusted accuracy of the model with each added variable.

To conduct the analysis, we relied on the R software, version 3.4.3, with dedicated packages such as “ROC” to assess the accuracy of the logistic models; the package “sjstats” was used to extract and test the statistical significance of the correlation between our predicted dichotomous variables, and the package “car” helped in assessing the variance inflation factors and test for multicollinearity.

## 3. Results

### 3.1. Sample Description

Our final sample consisted of 587 respondents, but only 563 surveys were considered valid. The overall sample was then split into the two relevant groups. The first group consisted of high school students and had 310 respondents (150—females and 160—males), while the second group included junior students in Medicine, and had 253 respondents (176—females and 77—males). Table 1 provides the description of the sample.

Table 2 presents the choices made by the respondents in each group across the four scenarios. While in the case of the high school students, the majority choose “Yes” to the first question in all scenarios, with medical students, choices significantly changed for the FD and MFD scenarios. The potential explanations and implications of this result are discussed in the Section 4.

We used the phi coefficient of correlation [49,50] to compute the association between our binary predictors within each scenario. As presented in Appendix A, the predictors have either statistically significant but moderate correlations with each other, or no significant correlations (as in the case of gender), so we can safely use them together as independent variables in our models.

### 3.2. The Logistic Regression Models

Our baseline model is built on the preliminary observation that 45% of the respondents in our sample were medical students, and 55% of the respondents were high school students. Therefore, when randomly choosing a respondent, the most likely event is that it will be a high school student. Such a baseline prediction has an extremely low accuracy of only 55%, which we aimed to improve by including the choices at each of the four scenarios as the main predictors after controlling for gender. We purposefully excluded age, as it classifies a respondent in one of the two groups with near certainty.

First, we randomly split our data into a training set containing 70% of the observations, and a test set including the remaining 30% of the respondents. We developed a prediction model on the training set, and then assessed its accuracy on the test set. Table 3 presents the coefficients of the logistic model on the train set.

Table A1 in Appendix A shows the correlations among our predictors and indicates a 66.7% correlation between FD and MFD. We therefore tested for multicollinearity using the variance inflation factors and found the VIF coefficients shown in Table 4. Although the VIF associated to MFD was close to 2, we still can consider that the correlation between MFD and FD did not harm the estimation [51,52,53].

Our results show that three out of the four scenarios were relevant in predicting the group: TD (ß_1_ = 0.862), MTD (ß_2_ = −0.916), and FD (ß_3_ = 1.049). In particular, the negative sign of the MTD coefficient shows that those who answered ‘no’ to this scenario were less likely to be medical students than those who answered ‘yes’. With the FD and TD, the situation is reversed: those who answered ‘no’ in these cases were in fact more likely to be medical students than those who chose ‘yes’.

Gender proves to be statistically significant in the sense that males were less likely to be medical students (ß_5_ = −0.707). This confirms the initial descriptive statistic that shows that 70% of the medical students were female. The accuracy of the model on the training set increased from its baseline performance of only 55%, reaching 72%. 

### 3.3. Cross Validation and Sensitivity Analysis

To rule out the risk of overfitting, we predicted the model on the test set and found an accuracy of prediction of 69%. The value is nearly as high as the accuracy of the model on the training set; therefore, we can admit that our model has a good prediction power outside the context on which it was created.

One last step in assessing model accuracy is a sensitivity analysis, which aims to explore the adjusted regression coefficients with each added variable. Table 5 shows that if we start with the control variable only, the accuracy of the model is 60.7%, lower than the accuracy of the overall model as presented in Table 3. As we add more variables, the accuracy of the model predicting the respondent’ group increases. In addition, the statistical significance of the predictors, along with their sign, are preserved. The models presented in Table 5 account for the entire dataset, and not only for the training set.

## 4. Discussion and Conclusions

The relevance of moral dilemmas in the field of professional ethics is widely acknowledged in the literature as a tool for both theoretical exploration and teaching. In their seminal book, Beauchamp and Childress [54] point out that despite a cross-millennial concern for ethics in medical sciences, the recent medical advancements pose new challenges which cannot be properly accounted for by the Hippocratic oath alone. For instance, principlism aims to offer a sound theory and decision-making framework for how healthcare practitioners ought to act. However, even when such a framework is available, they admit that some ethical issues pose serious theoretical and practical challenges. Thus, it is agreed upon that such authentic moral dilemmas are subject to reasonable disagreement [54], but also an opportunity to further reflect on the adaptive rationality involved in complex decisions. In this vein, it is noteworthy to consider that moral dilemmas are characterized by the fact that two or more ethical principles, values, duties, or obligations are conflicting with one another. Sometimes moral dilemmas are ‘solved’ by a top-down consensus, such as specific medical procedures and laws to help and guide doctors. For example, in case of a natural disaster, earthquake, or firestorm, when doctors cannot help everybody who needs medical attention, they must conduct a triage based on certain criteria. However, the laws can also be incomplete (e.g., they are not necessarily the result of an evidence-based process or even a comprehensive deliberation process), or they simply vary significantly from one country to another, especially on controversial topics such as abortion [55], euthanasia [56], withdrawal, or withholding treatment [57]. Therefore, it is natural that the rules enforced as laws and procedures do not address all the medical dilemmas that doctors encounter in their daily practice. Moreso, it may be that sometimes they are purposely designed to offer limited freedom for medical practitioners to adjust their judgment in different contexts. Mirroring this view, Kushner and Thomasma [58] developed a handbook based exclusively on dilemmas as a methodological instrument of conducting trainings for healthcare personnel.

Another important aspect is that philosophical dilemmas differ from the real-life dilemmas in the sense that they eliminate uncertainty from the presented situation. Although precise facts are extremely relevant for ethics, they can also have the drawback of easily derailing people from an ethical discussion as a central concern, or they may activate various emotional cues and implicitly behavioral biases (e.g., the identifiable victim effect), and involve people personally. Thus, the participants are asked to imagine a possible world where everything happens exactly as we state and then the answers of the participants are analyzed against this control set of environmental characteristics. As mentioned in the Section 1, we followed this validated path and the sacrificial dilemmas from our study were the ones extensively used by moral psychologists to examine different moral judgments in experimental settings, although they were initially designed for purely philosophical purposes.

In consequence, we are confident that the dilemmas we deployed reflect some of the moral decisions (e.g., considerable similitudes in the existing constraints and resources, thus in the potential scarcity mindset) professional medical staff are trained to make on a normal basis. Our findings show that the moral judgments in the provided scenarios act as relevant predictors for the group of medical students: those answering “no” to the footbridge dilemma and to the trolley dilemma were more likely to be medical students. This is useful evidence not because we are not trying to advance any normative claim when it comes to the possible “solutions” for the dilemmas. Engaging with the philosophical and legal complications of sacrificial scenarios, although highly enticing and challenging as a topic, is beyond the scope of our paper. On the contrary, our aim is more modest but also more practical: to emphasize a descriptive reality that happens also in this particular context. Namely, that individuals exhibit systematic biases determined by their own psychological profile, augmented by education, training, and social exposure. Further, we formalize and we describe quantitatively the extent to which preferences elicited through the moral dilemmas procedure predict the selection bias among medical students.

While arguably the ‘real-life’ scenarios are not as extreme or proportionally equivalent to the ones in the dilemmas, healthcare professionals and organizations are routinely faced with the challenge of maximizing the amount of good they can do with limited resources [59]. If in a situation where a limited number of medical supplies could either be used to save the lives of five patients in a less severe condition or the life of just one patient in a very severe condition, probably most doctors would agree that, caeteris paribus, the first course of action is morally justifiable [60]. This feature of the ethical culture in medicine [61] goes hand in hand with the typically ‘utilitarian’ answers we have received from junior medical students in both the TD and MTD scenarios (see Table 2). Medical students would choose to save five persons in a proportion of 69% in TD, and 56% in MTD. Similar ‘utilitarian’ answers were given by high school students in TD (86%) and MTD (61%).

On the other hand, in FD and MFD, medical students refused to sacrifice the person on the bridge to save five others (FD: 70%—No; MFD: 61%—No), while the high school students kept their seemingly ‘utilitarian’ response (FD: 57%—Yes; MFD: 61%—Yes). We interpret the divergence in analogy with a situation where it would be necessary for a doctor to kill a patient to save five others (by harvesting organs, for example). There is a consensus in the profession that such a course of action is forbidden by medical ethics and law alike. In such a scenario, the moral stance ceases to be crudely utilitarian, and it reflects the Hippocratic injunction for doctors to, first and foremost, do no harm (‘primum non nocere’). It would not be implausible to expect the same doctors who choose to “save” five persons in TD to refuse ‘killing’ one person in FD. The fact that first-year students in medicine already make these moral judgments seems to indicate that, even before any formal or informal ethical training in a medical school, they tend to intuitively share some of the key insights from medical ethics.

Although the current data and design are not sufficient to support conclusively the hypothesis of a moral self-selection effect in the academic choice of high school students (we do not know if the selection effect is specific to medical students, if the high school participants’ preferences for college influenced the results, or if the dilemmas offer enough control to efficiently test moral judgments and rule out a maturation effect), we believe that some of our results in particular are encouraging and warrant further testing. On the one hand, how our participants responded in the moral dilemmas plays a statistically significant role in differentiating the medical students from the rest of the students. On the other hand, the contribution of these dilemmas to the overall accuracy of our models seems to be sizeable. As Table 5 shows, if only the control variable is considered, the accuracy of the prediction is 60.7%. With the respondents’ decisions on the dilemmas, the accuracy increases to 71.1%, which proves their contribution in predicting the group (either a high school student, or a medical student). By using a cross-validation procedure, we found that the prediction model has a good performance not only on the set it was built on, but also on a different set.

What makes our result even more interesting is that the predictors we considered improved the accuracy from an initial baseline value of 55% to 71%. Put in different words, in the very first instance, the prediction power in assigning a respondent to a certain group was nearly as good as flipping a coin. By considering the way these respondents choose to answer the “factual” question in the four scenarios, and only gender as a control variable, we improved the level of accuracy by 17%.

Our results are promising and seem to highlight moral judgment as a significant predictor in our sample for choosing medicine as a bachelor, but we agree that further research would be needed to claim that a kind of moral self-selection effect is generally present in the choice of a bachelor program. Either longitudinal studies exploring how moral judgments made by high school students in different grades correlate with their prospective college preferences or similar studies conducted on first-year students in other scientific fields than medicine should bring valuable insights [62]. In our view, a plausible hypothesis for further study is that, if such a self-selection effect exists, it would be stronger in fields with a well-established and rather unified ethical culture such as medicine, religion [63], and social work [64], and weaker in the fields with less spelled out ethical cultures. 

We also admit that the intuitive responses to sacrificial dilemmas draw at best an incomplete picture of the comprehensive moral outlook that a person might hold. Some authors [65,66,67] raised several concerns regarding how effective sacrificial dilemmas are as empirical tests to measure moral judgments and what lay people’s intuitive explicit responses tell us, from a normative point of view, about moral judgments in general. Moral psychologists use a much wider array of tools in order to identify both explicit and implicit moral evaluations, ranging from a plethora of more or less dramatic dilemmas and scenarios to neuroimaging [42] or quick association tests [68]. A comprehensive description of the set of moral judgments entertained by first-year medical students should deploy more of these tools and integrate the results of the existing literature on empathy. However, we did not seek to reach such a description; our significantly more limited claim is just that, in the scenarios that we used, the junior medical students made the kind of moral judgments that one would expect from a trained and experienced medical professional. This result should be interpreted as supporting the idea that we need to develop new studies and methods to observe whether our hypothesis that the vocational preference for pursuing a medical bachelor’s degree correlates with specific moral judgments. We believe that further research in this direction might also hold some promising implications for higher education research and policies.

There is a wide agreement that empathy very often modulates our moral decisions and behavior; empathy scales should be used alongside sacrificial dilemmas to measure morality in follow up studies for checking whether they could predict our hypothesis that morality is a self-selection effect for following a medical career.

Another important limitation of our study which should be addressed in future experimental designs is to test if moral judgments in sacrificial dilemmas are a predictor for following a career only in medicine or also in other intuitively related professions such as law, police, or military. It sounds plausible to formulate hypotheses based on a comparison between different career paths and see whether there is a difference between them from the point of view of the moral judgments they are inclined to make. The responses to sacrificial dilemmas alone are not sufficient to establish a causal relation between the decision to enroll to the medical school and the inherent moral judgments made by prospective medical students.

Our data encourage research in this direction which we believe that it could have at least two major implications. First, it could have clear implications in the field of academic choice. Expanding academic choice models to include moral judgments, at least for the fields with a strong ethical culture, can further enrich the existing heterogenous theoretical framework [69,70,71]. Additionally, from an academic policy perspective, it can provide a useful tool for improving future student satisfaction with their career choice (if this is screened appropriately in the selection process). This benefits both the individuals, through the prospective positive impact on their academic motivation and performance, and the institutions, in terms of retention rates.

A second set of implications regards the case of medical education, especially when it comes to the ethical training of future doctors. Teachers usually decide to approach such courses with the idea that one of their main roles is to instill a certain set of moral values. However, if, as our results suggest, students already make the kind of judgments that we associate with the core values in medical ethics when they choose to enroll in medical school, then the common approach risks often ending up “preaching to the choir”. Instead of focusing on shaping moral intuitions and judgments, an alternative approach for developing the content of ethics courses in medical schools [72] would rather favor putting the already existing moral intuitions to work and training moral reasoning (and other non-technical skills [73,74]) through the exercise of ethical analysis of difficult cases [75]. In a nutshell, this could be a leading direction for potential reform on developing medical professionalism in future doctors [29,76,77]. This is globally relevant but even moreso for Romania, in light of the current medical exodus experienced by the profession [78,79].

In conclusion, considering the limited research on the determinants of academic choice, this paper encourages testing novel proposals on how to improve the situation of medical professionals from a more comprehensive perspective and at an early stage of selection [80], beyond the existing policies focused mostly on the end results and financial incentives [81]. This means that an exploratory study such as ours is not necessarily built on an already locally tested Romanian university choice model, but rather draws on the existing experiences of other countries. While we acknowledge the need to close this gap by incorporating more factors into the analysis, we also advocate for a wider array of evidence-based measures in critical areas such as health sciences.

## Figures and Tables

**Table 1:** Descriptive statistics—control variables.

Variable	Descriptive Statistics (n = 563)				
Numerical variable	Min	Median	Mean	Max	SD
Age (Group 1)	13	15	15.57	20	1.39
Age (Group 2)	18	19	19.04	25	0.79
Categorical variables	Proportion				
Gender (Group 1)					
Female	48%				
Male	52%				
Gender (Group 2)					
Female	70%				
Male	30%				

**Table 2:** Descriptive statistics—respondents’ choice by group.

Scenario	High School Students (n = 310)	Medical Students (n = 253)
TD model	86.0% (Yes)	69.0% (Yes)
	14% (No)	31% (No)
MTD model	61.0% (Yes)	56.0% (Yes)
	39% No	44% (No)
FD model	57.0% (Yes)	70.0% (Yes)
	43% (No)	30% (No)
MFD model	61.0% (Yes)	61.0% (Yes)
	39% (No)	39% (No)

**Table 3:** Estimated coefficients of the logistic model predicting the respondents’ group, fitted on the training set (standard errors in parentheses).

Model	Group (Medical Versus Non-Medical Students) (n = 394)
Intercept	−0.614 ** (0.197)
TD factual Yes No	*Reference* 0.862 ** (0.318)
FD factual Yes No	*Reference* 1.049 *** (0.297)
MTD factual Yes No	*Reference* −0.916 ** (0.292)
MFD factual Yes No	*Reference* 0.505 (0.307)
Gender Female Male	*Reference* −0.707 ** (0.229)
*AUC*	*0.720*

***—*p* value < 0.001; **—*p* value < 0.01.

**Table 4:** The variance inflation factors for our predictors.

Variable	TD	FD	MTD	MFD	Gender
VIF	1.443	1.751	1.706	1.949	1.009

**Table 5:** The change in the predictive accuracy of the logistic models as we added variables.

Model (n = 563)	Group	Group	Group	Group	Group
Intercept	1.064 *** (0.262)	−0.491 ** (0.156)	−1.213 ** (0.445)	−0.935 * (0.458)	−1.019 * (0.463)
FD factual Yes No	-	*Reference* 1.137 *** (0.183)	*Reference* 0.987 *** (0.194)	*Reference* 1.125 *** (0.204)	*Reference* 0.934 *** (0.248)
TD factual Yes No	-	-	*Reference* 0.528 * (0.234)	*Reference* 0.842 ** (0.263)	*Reference* 0.825 ** (0.263)
MTD factual Yes No	-	-	*-*	*Reference* −0.615 ** (0.226)	*Reference* −0.693 ** (0.234)
MFD factual Yes No	-	-	*-*	-	*Reference* 0.337 (0.255)
Gender Female Male	*Reference* −0.904 *** (0.178)	*Reference* −0.895 *** (0.185)	*Reference* −0.843 *** (0.187)	*Reference* −0.850 *** (0.188)	*Reference* −0.845 *** (0.189)
*AUC*	*0.607*	*0.687*	*0.698*	*0.708*	*0.711*

***—*p* value < 0.001; **—*p* value < 0.01; *—*p* value < 0.05; .—*p* value < 0.10.

## Data Availability

The dataset and survey material supporting this article have been uploaded on the Open Science Framework: https://osf.io/k4enx/?view_only=8cc6a8f4e20544e6974763f8c1a7c1c5.

## Appendix A

**Table A1:** Correlations between the categorical predictors.

	TD Factual	MTD Factual	FD Factual	MFD Factual	Gender
TD factual	1	0.497 ***	0.362 ***	0.362 ***	0.151 ***
MTD factual		1	0.356 ***	0.446 ***	0.069
FD factual			1	0.667 ***	0.067
MFD factual				1	0.096 *
Gender					1

***—*p* value < 0.001; *—*p* value < 0.05; .—*p* value < 0.10.

## Appendix B

Modified trolley dilemma (MTD)

*“You are near a railway track when you suddenly see a runaway trolley. Further down its course five, persons are tied up to the tracks. If you choose to do nothing, all five will be killed by the trolley. Luckily, there is a switch near you. If you activate the switch the trolley will be redirected to a secondary line where, just a few feet from you, a person is tied up and would be run over by the trolley. You know these are your only two options.”*

Modified footbridge dilemma (MFD)

*“You work for the railway traffic control, in a building a few miles away from a footbridge that crosses a railway. While you were drinking your coffee you see, on your monitor, that a runaway trolley is heading towards the footbridge and threatens the life of five people who are tied up to the tracks. The only thing you can do, in order to save their lives, is to push a button which opens a hatch under the bridge. A fat man standing on the bridge will fall through the hatch and die, but in the process s/he also stops the trolley hence saving 5 lives.”*
